# Relative transmissibility of hand, foot and mouth disease from male to female individuals

**DOI:** 10.1017/S0950268819001729

**Published:** 2019-10-07

**Authors:** Yuxue Liao, Yaqing He, Yan Lu, Hong Yang, Yanhua Su, Yi-Chen Chiang, Benhua Zhao, Huawei Xiong, Tianmu Chen

**Affiliations:** 1 Shenzhen Center for Disease Control and Prevention, Shenzhen, Guangdong Province, People's Republic of China; 2 State Key Laboratory of Molecular Vaccinology and Molecular Diagnostics, School of Public Health, Xiamen University, Xiamen, Fujian Province, People's Republic of China

**Keywords:** Hand, foot and mouth disease, intersex transmission, mathematical model, relative transmissibility

## Abstract

Hand, foot and mouth disease (HFMD) has spread widely and leads to high disease burden in many countries. However, relative transmissibility from male to female individuals remains unclear. HFMD surveillance database was built in Shenzhen City from 2013 to 2017. An intersex transmission susceptible–infectious–recovered model was developed to calculate the transmission relative rate among male individuals, among female individuals, from male to female and from female to male. Two indicators, ratio of transmission relative rate (*R*_*β*_) and relative transmissibility index (RTI), were developed to assess the relative transmissibility of male *vs*. female. During the study period, 270 347 HFMD cases were reported in the city, among which 16 were death cases with a fatality of 0.0059%. Reported incidence of total cases, male cases and female cases was 0.0057 (range: 0.0036–0.0058), 0.0052 (range: 0.0032–0.0053) and 0.0044 (range: 0.0026–0.0047), respectively. The difference was statistically significant between male and female (*t* = 3.046, *P* = 0.002). *R*_*β*_ of male *vs.* female, female *vs.* female, from female to male *vs.* female and from male to female *vs.* female was 7.69, 1.00, 1.74 and 7.13, respectively. RTI of male *vs.* female, female *vs.* female, from female to male *vs.* female and from male to female *vs.* female was 3.08, 1.00, 1.88 and 1.43, respectively. Transmissibility of HFMD is different between male and female individuals. Male cases seem to be more transmissible than female.

## Introduction

Hand, foot and mouth disease (HFMD) is an important infectious disease and leads to high disease burden in many countries [1–6]. There are over 20 types of enteroviruses leading to HFMD [1]. The main pathogens of the disease are Enterovirus 71 (EV71) and Coxsackievirus A16 (CV-A16). The complexity of the pathogens leads to difficulty in controlling the disease. Therefore, it is essential to understand the transmissibility of HFMD. Understanding the transmissibility of an infectious disease could help health department to forecast the attack rate and assess the effectiveness of countermeasures to contain the spread of the disease [7–12].

Several mathematical models have been developed to calculate the transmissibility of HFMD, and the results of these research studies showed that the transmissibility of HFMD has a wide-span range. The estimated basic reproduction number (*R*_0_) was 1.44 in Bangkok, Thailand, 2016 [2]. Results of a mathematical model study showed that the average *R*_0_ of three different strains of EV71 from Japan, Malaysia and Thailand were 37.35 ± 8.99, 8.37 ± 0.82 and 6.75 ± 0.16, respectively [13]. Another study showed that the median *R*_0_ of CV-A6, CV-A16 and EV-A71 in Singapore was estimated to be 5.04 (interquartile range (IQR) 3.57–5.16), 2.42 (IQR 1.85–3.36) and 3.50 (IQR 2.36–4.53), respectively [14]. Wang *et al*. [15] employed a susceptible–infectious–recovered (SIR) model to calculate the transmissibility of HFMD in 2008 and 2009 in China, and found that the effective reproductive number had a median of 1.4 (range: 1.4–1.6) in spring and stayed below 1.2 in other seasons. Takahashi *et al*. [16] found that the transmissibility of the disease was much higher from 2009 to 2013 in China. The *R*_0_ was 26.63 (IQR: 23.14–30.40) for Enterovirus 71 (EV71) and 27.13 (IQR: 23.15–31.34) for Coxsackievirus A16 (CV-A16) estimated by a time series SIR (TSIR) model [16]. Calculated the case-based data from 2009 to 2012 by the TSIR model, the median reproductive number of HFMD was 4.62 (IQR: 3.91–5.82) in Guangdong Province and 3.11 (IQR: 2.44–4.43) in Shenzhen City, respectively [17]. Undoubtedly, these research studies about the transmissibility of different pathogens in different areas have provided much epidemiological information for understanding and controlling HFMD.

However, significance difference in the incidence exists between male and female [18–20]. Wang *et al*. [15] found that the attack rate of male was higher than that of female in 2008 and 2009 in China. The significant gender differences reveals that the transmissibility of male might different to that of female. Unfortunately, the relative transmissibility from male to female individuals remains unclear. In this study, we first built case-based epidemiological data of reported HFMD cases from 2013 to 2017 in Shenzhen City, Guangdong Province, China. An intersex transmission SIR model was then developed according to the natural history and the intersex transmission mechanism of the disease to fit the epidemiological data. Finally we developed a relative transmissibility index (RTI) calculated by the model to assess the relative transmissibility of male *vs*. female.

## Materials and methods

### Data collection

A dataset of reported HFMD cases (clinically diagnosed cases and confirmed cases) and population information, collected from the Chinese Disease Control and Prevention Information System, was built in Shenzhen City from February 2013 to December 2017. The illness onset date and sex (male or female) of each case were collected. The population information included number of male and female individuals, birth rate and death rate of the population. The city which locates in the south China is a large city in Guangdong Province. It has a population of more than 12 million inhabitants and has a median birth rate of 18.40 per 1000 people (range: 17.48 per 1000 people to 19.94 per 1000 people) and median death rate of 6.72 per 1000 people (range: 6.63 per 1000 people to 9.72 per 1000 people) from 2013 to 2017.

### The intersex transmission model

**Fig. 1.**
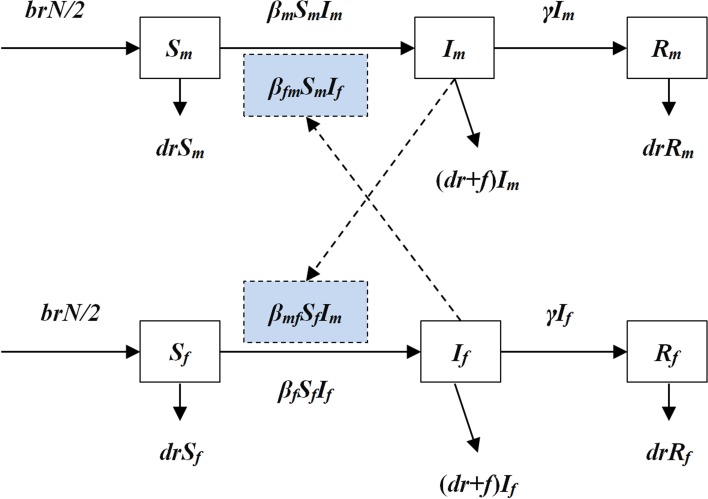
The diagram of intersex transmission SIR model of HFMD.

An intersex transmission SIR model was developed according to the natural history of HFMD and the mechanism of the transmission between male and female individuals (Fig. 1).

In the model, we assumed that: (a) transmission relative rate among male and female individuals was *β*_m_ and *β*_f_, respectively and (b) transmission relative rate from male to female was *β*_mf_ and from female to male was *β*_fm_. Therefore, the transmission model was shown as follows:
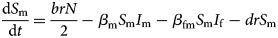

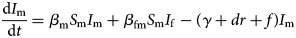

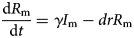

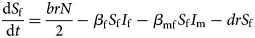

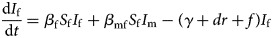

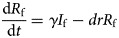

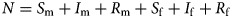


In the above equations, *S*, *I* and *R* refer to susceptible individuals, infectious individuals and recovered individuals, respectively. The subscripts m and f refer to male and female. *N* refers to the number of the whole population. Parameters *br*, *dr*, *f*, *β* and *γ* refer to natural birth rate of the population, death rate of the population, fatality of HFMD, transmission relative rate and recovered relative rate, respectively.

### Parameter estimation

There were eight parameters (*β*_m_, *β*_f_, *β*_mf_, *β*_fm_, *br*, *dr*, *f* and *γ*) in the model (Table 1). Parameters *br*, *dr* and *f* were calculated from the collected data. According to the yearly values of *br* and *dr*, we calculated the weekly values of the two parameters. Therefore, the weekly value of *br* and *dr* was 0.000352 (range: 0.000330–0.000383) and 0.0000129 (range: 0.0000127–0.0000187), respectively. According to the published study [16, 17], the infectious period of HFMD was about 2 weeks, therefore *γ* = 0.5. The collected data of reported HFMD cases were employed to fit the SIR model to calculate *β*_m_, *β*_f_, *β*_mf_ and *β*_fm_ in each epidemic cycle.

**Table 1.** Parameter definitions and values

Parameter	Description	Unit	Value	Range	Method
*β*_m_	Transmission relative rate among male individuals	1	See text	0–1	Curve fitting
*β*_f_	Transmission relative rate among female individuals	1	See text	0–1	Curve fitting
*β*_fm_	Transmission relative rate from female to male	1	See text	0–1	Curve fitting
*β*_mf_	Transmission relative rate from male to female	1	See text	0–1	Curve fitting
*γ*	Recovered relative rate	per day	0.5	0–1	14, 15
*br*	Birth rate of the population	1	3.52 × 10^−4^	3.30 × 10^−4^–3.83 × 10^−4^	Analysis on the reported data
*dr*	Death rate of the population	1	1.29 × 10^−5^	1.27 × 10^−5^–1.87 × 10^−5^	Analysis on the reported data
*f*	Fatality of the disease	1	5.90 × 10^−5^	0–1	Analysis on the reported data

### Indicators to assess the relative transmissibility of male *vs.* female

Two indicators, ratio of transmission relative rate (*R*_*β*_) and RTI, were developed to assess the relative transmissibility of male *vs*. female. Let *i* = 1, 2, 3 and 4 refers to transmissibility among male individuals, among female individuals, from female to male and from male to female, respectively. The subscript *j* refers to the compared group, and was set as transmissibility among female individuals in this study. Therefore, four scenarios were simulated as M *vs.* F, F *vs.* F, FM *vs.* F and MF *vs.* F, where M, F, FM and MF refer to male, female, from female to male and from male to female, respectively. The equations to calculate *R*_*β*_ and RTI were shown as follows:
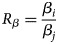

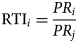

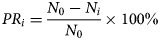


In the above equations, *PR*_*i*_, *N*_0_ and *N*_*i*_ refer to percentage of reduction under different intervention scenarios (*β*_m_ = 0, *β*_f_ = 0, *β*_fm_ = 0 and *β*_mf_ = 0), number of cases under the condition that no intervention was adopted and number of cases under the condition that four intervention scenarios (*β*_m_ = 0, *β*_f_ = 0, *β*_fm_ = 0 and *β*_mf_ = 0) were simulated, respectively.

### Statistical analysis

Berkeley Madonna 8.3.18 (developed by Robert Macey and George Oster of the University of California at Berkeley. Copyright ©1993–2001 Robert I. Macey & George F. Oster) was employed to run the model and least root mean square was adopted to assess goodness of fit. SPSS 13.0 (IBM Corp., Armonk, NY, USA) was employed to run the *t* test between male and female and Kruskal–Wallis test among *β*_m_, *β*_f_, *β*_mf_ and *β*_fm_.

## Results

### Epidemiological characteristics of reported HFMD cases

**Fig. 2.**
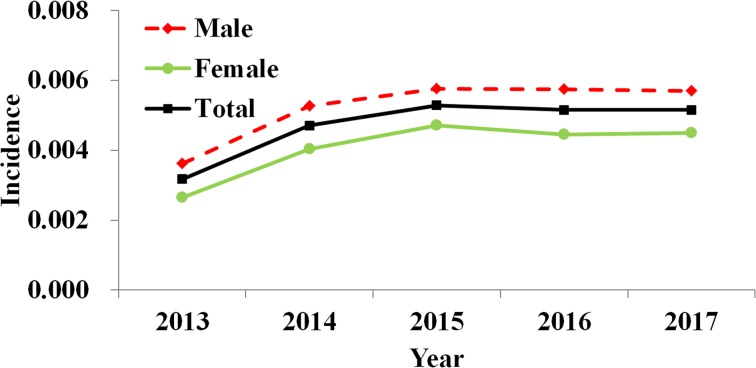
Yearly reported incidence of HFMD in Shenzhen City, 2013 to 2017.

From 2013 to 2017, 270 347 HFMD cases (including 162 757 male cases and 107 590 female cases) were reported in Shenzhen City, among which 16 were death cases with a fatality of 0.0059%. Reported incidence of total cases, male cases and female cases increased yearly with a median value of 0.0057 (range: 0.0036–0.0058), 0.0052 (range: 0.0032–0.0053) and 0.0044 (range: 0.0026–0.0047), respectively (Fig. 2).

**Fig. 3.**
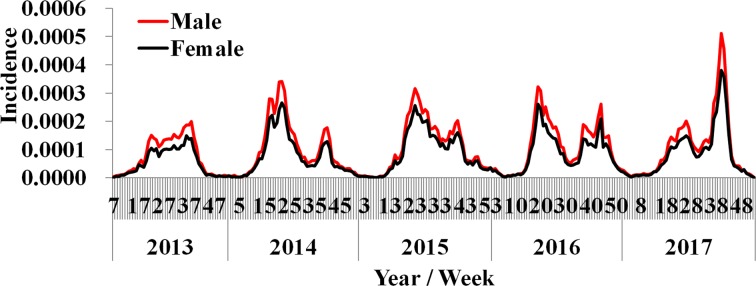
Weekly reported incidence of HFMD in Shenzhen City from week 7, 2013 to week 53, 2017.

By analysing the weekly reported data, almost two epidemic cycles were observed at the turn of seasons from spring to summer and from summer to autumn in a year. These cycles were observed from both male and female cases. However, the reported incidence of male cases was slightly higher than female (Fig. 3). The difference of weekly incidence was statistically significant between male and female (*t* = 3.046, *P* = 0.002).

### Curve fitting results

**Fig. 4.**
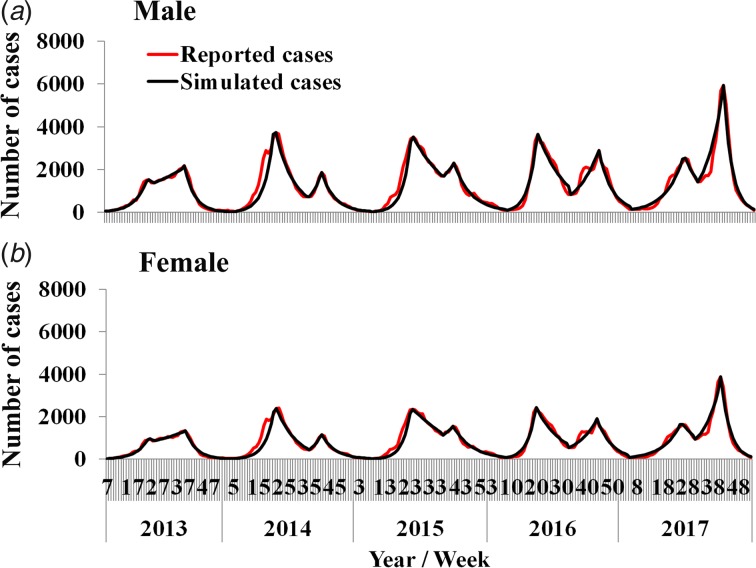
Curve fitting results run by the intersex transmission SIR model to weekly reported HFMD cases.

Results of curve fitting showed that the SIR model fitted the data well (Fig. 4). Four *β* values were calculated in a year, because there were two epidemic cycles in a year and ascending period and descending period of an epidemic cycle had different *β* values. All the values inner and between male and female individuals are shown in Table 2. The median value of *β*_m_, *β*_f_, *β*_mf_ and *β*_fm_ was 4.78 × 10^−8^ (range: 1.09 × 10^−13^–1.23 × 10^−7^), 6.21 × 10^−9^ (range: 2.57 × 10^−17^–1.12 × 10^−7^), 1.08 × 10^−8^ (range: 1.99 × 10^−14^–2.19 × 10^−7^) and 4.43 × 10^−8^ (range: 9.53 × 10^−15^–9.89 × 10^−8^), respectively. The results of Kruskal–Wallis test showed that the difference among *β*_m_, *β*_f_, *β*_mf_ and *β*_fm_ was statistically significant (*χ*^2^ = 7.938, *P* = 0.047). Therefore, *R*_*β*_ of M *vs.* F, F *vs.* F, FM *vs.* F and MF *vs.* F was 7.69, 1.00, 1.74 and 7.13, respectively.

**Table 2.** Transmission relative rate in epidemic cycle from 2013 to 2017 in Shenzhen City

Year	Epidemic cycle	*β*_m_	*β*_f_	*β*_fm_	*β*_mf_
2013	Cycle 1	1.23 × 10^−7^	2.57 × 10^−17^	2.64 × 10^−9^	9.11 × 10^−8^
		6.80 × 10^−8^	9.01 × 10^−8^	1.39 × 10^−8^	1.66 × 10^−12^
	Cycle 2	7.18 × 10^−8^	9.99 × 10^−16^	3.48 × 10^−8^	6.80 × 10^−8^
		4.36 × 10^−8^	2.72 × 10^−9^	7.73 × 10^−14^	3.32 × 10^−8^
2014	Cycle 1	1.09 × 10^−13^	1.73 × 10^−8^	2.19 × 10^−7^	8.52 × 10^−8^
		3.28 × 10^−8^	3.15 × 10^−14^	4.41 × 10^−8^	5.11 × 10^−8^
	Cycle 2	2.34 × 10^−13^	7.62 × 10^−8^	1.88 × 10^−7^	4.15 × 10^−8^
		3.19 × 10^−8^	6.40 × 10^−8^	3.17 × 10^−8^	2.00 × 10^−10^
2015	Cycle 1	3.82 × 10^−13^	5.25 × 10^−9^	2.02 × 10^−7^	9.89 × 10^−8^
		7.07 × 10^−8^	3.42 × 10^−8^	7.04 × 10^−14^	3.62 × 10^−8^
	Cycle 2	7.99 × 10^−8^	4.04 × 10^−8^	1.98 × 10^−8^	4.71 × 10^−8^
		4.77 × 10^−8^	7.04 × 10^−8^	1.36 × 10^−8^	9.53 × 10^−15^
2016	Cycle 1	1.20 × 10^−7^	1.92 × 10^−9^	8.01 × 10^−9^	9.46 × 10^−8^
		4.50 × 10^−8^	7.35 × 10^−8^	2.91 × 10^−8^	1.54 × 10^−14^
	Cycle 2	9.52 × 10^−8^	1.12 × 10^−7^	9.52 × 10^−13^	4.94 × 10^−14^
		4.78 × 10^−8^	7.17 × 10^−9^	2.35 × 10^−14^	3.68 × 10^−8^
2017	Cycle 1	9.49 × 10^−8^	1.21 × 10^−9^	3.25 × 10^−14^	7.26 × 10^−8^
		5.73 × 10^−8^	3.73 × 10^−14^	4.22 × 10^−14^	4.78 × 10^−8^
	Cycle 2	9.44 × 10^−9^	1.30 × 10^−14^	1.99 × 10^−14^	6.93 × 10^−8^
		2.85 × 10^−8^	2.68 × 10^−14^	2.31 × 10^−10^	2.77 × 10^−8^

### Relative transmissibility

The simulation results showed that the 5-year-average number of cases was 54 026 among which 32 524 were male cases and 21 502 were female cases.

**Fig. 5.**
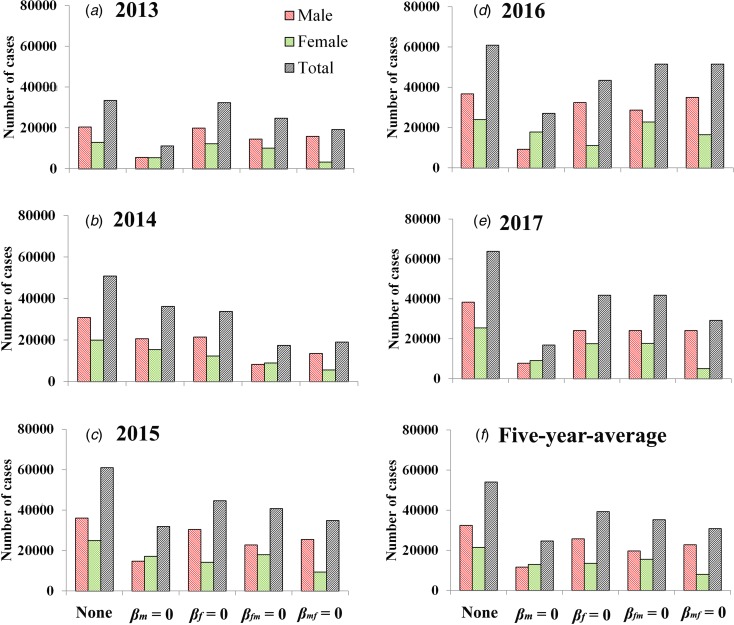
Reduction of cases under the different conditions (none, *β*_m_ = 0, *β*_f_ = 0, *β*_fm_ = 0 and *β*_mf_ = 0). (A–E) Scenarios in 2013 to 2017; (F) results of 5-year-average value. None refers to no intervention implemented.

If we set *β*_m_ = 0, the 5-year-average value of total cases was reduced 54.27% [(54 026 − 24 706)/54 026 × 100%] and male and female cases was reduced 64.22% [(32 524 − 11 638)/32 524 × 100%] and 39.22% [(21 502 − 13 069)/21 502 × 100%], respectively. If we set *β*_f_ = 0, the 5-year-average value of total cases was reduced 27.31% [(54 026 − 39 269)/54 026 × 100%] and male and female cases was reduced 20.87% [(32 524 − 25 735)/32 524 × 100%] and 37.06% [(21 502 − 13 534)/21 502 × 100%], respectively. If we set *β*_fm_ = 0, the 5-year-average value of total cases was reduced 34.69% [(54 026 − 35 284)/54 026 × 100%] and male and female cases was reduced 39.24% [(32 524 − 19 760)/32 524 × 100%] and 27.80% [(21 502 − 15 524)/21 502 × 100%], respectively. If we set *β*_mf_ = 0, the 5-year-average value of total cases was reduced 42.94% [(54 026 − 30 830)/54 026 × 100%] and male and female cases was reduced 29.85% [(32 524 − 22 816)/32 524 × 100%] and 62.73% [(21 502 − 8014)/21 502 × 100%], respectively. Similar results were observed in 2013 and 2017, except in 2014–2016 (Fig. 5 and Table 3).

**Table 3.** PR (%) in the four scenarios (*β*_m_ = 0, *β*_f_ = 0, *β*_fm_ = 0 and *β*_mf_ = 0) from 2013 to 2017 in Shenzhen City

Year	Sex	*β*_m_ = 0	*β*_f_ = 0	*β*_fm_ = 0	*β*_mf_ = 0
2013	Male	72.36	2.42	28.51	22.61
	Female	57.26	4.37	21.53	73.81
	Total	98.97	4.73	38.40	63.11
2014	Male	32.86	30.15	72.73	56.17
	Female	22.44	38.34	55.01	71.93
	Total	28.76	33.37	65.76	62.37
2015	Male	59.12	15.58	36.67	29.53
	Female	30.99	43.10	27.99	62.12
	Total	47.62	26.83	33.12	42.86
2016	Male	74.82	11.96	22.11	4.83
	Female	25.64	53.70	5.09	31.26
	Total	55.40	28.44	15.39	15.26
2017	Male	79.68	36.87	36.95	36.91
	Female	64.01	31.11	30.88	80.10
	Total	73.41	34.57	34.52	54.18
Average	Male	64.22	20.87	39.24	29.85
	Female	39.22	37.06	27.80	62.73
	Total	54.27	27.31	34.69	42.94

When we focus on the 5-year-average male cases, RTI of M *vs.* F, F *vs.* F, FM *vs.* F and MF *vs.* F was 3.08, 1.00, 1.88 and 1.43, respectively. When we focus on the 5-year-average female cases, RTI of M *vs.* F, F *vs.* F, FM *vs.* F and MF *vs.* F was 1.06, 1.00, 0.75 and 1.69, respectively. When we focus on the 5-year-average total cases, RTI of M *vs.* F, F *vs.* F, FM *vs.* F and MF *vs.* F was 1.99, 1.00, 1.27 and 1.57, respectively. Similar results were observed in 2013 and 2017, except in 2014–2016 (Table 4).

**Table 4.** RTI in the four scenarios (M *vs.* F, F *vs.* F, FM *vs.* F and MF *vs.* F) from 2013 to 2017 in Shenzhen City

Year	Sex	M *vs.* F	F *vs.* F	FM *vs.* F	MF *vs.* F
2013	Male	29.85	1.00	11.76	9.33
	Female	13.09	1.00	4.92	16.88
	Total	20.93	1.00	8.12	13.35
2014	Male	1.09	1.00	2.41	1.86
	Female	0.59	1.00	1.43	1.88
	Total	0.86	1.00	1.97	1.87
2015	Male	3.80	1.00	2.35	1.90
	Female	0.72	1.00	0.65	1.44
	Total	1.77	1.00	1.23	1.60
2016	Male	6.26	1.00	1.85	0.40
	Female	0.48	1.00	0.09	0.58
	Total	1.95	1.00	0.54	0.54
2017	Male	2.16	1.00	1.00	1.00
	Female	2.06	1.00	0.99	2.57
	Total	2.12	1.00	1.00	1.57
Average	Male	3.08	1.00	1.88	1.43
	Female	1.06	1.00	0.75	1.69
	Total	1.99	1.00	1.27	1.57

M, male; F, female; FM, from female to male; MF, from male to female.

## Discussion

Significant difference of HFMD incidence between male and female is commonly observed by the descriptive epidemiology method [20–23]. We assumed that this phenomenon is attributed to the different transmissibility among male and female individuals. In this study, the HFMD incidence of male was slightly higher than that of female in Shenzhen City, although the difference value was lower than the published data [15]. To verify our hypothesis, we developed an intersex transmission SIR model to explore the difference first. Our simulation results showed that the value of the transmission relative rate among male, among female, from male to female and from female to male was different, and they have the following order: *β*_m_ > *β*_mf_ > *β*_fm_ > *β*_f_. Therefore, the values of *R*_*β*_ have the following order: M *vs.* F > MF *vs.* F > FM *vs.* F > F *vs.* F.

Considering that *β* is a process parameter, it plays the role of transmission force behind the phenomenon. To make the outcomes more direct, we simulated several ‘knockout’ scenarios (*β*_m_ = 0, *β*_mf_ = 0, *β*_fm_ = 0 and *β*_f_ = 0) orderly. The results of the simulation showed that the values of RTI have the following order: M *vs.* F > MF *vs.* F > FM *vs.* F > F *vs.* F. This order is the same as that of *R*_*β*_. These findings revealed that male individuals are more transmissible than female individuals. Therefore, the different transmissibility between male and female is the reason of the significance of gender distribution.

Published research showed that most HFMD cases have an age lower than 5 years especially lower than 3 years [15, 18, 19, 24]. A system review showed that being male is a risk factor for both mild and severe HFMD [25]. Their findings suggest that boys are more likely to develop symptoms, more involved in propagation of outbreaks or more likely to be brought for medical care than girls [25]. Our results show that the values of *β* among male and from male to female were higher than those among female and from female to male. To our knowledge, boy is more active than girl. The daily contact rate among boys, from boy to girl and between boy and environment is higher than that of girl. These differences might lead to the higher values of *β* among male and from male to female. However, the value of *β* might be affected by multifactor including behaviour of individuals and environment. More research might be needed to explore the multifactorial interaction.

Of note, there is a limitation that the skewed distribution of age was not considered in our study. The relative transmissibility might be different at different age groups. However, to explore the age-specific relative transmissibility, more complex model and age distribution data are needed in the future.

## Conclusion

The HFMD incidence of male is higher than that of female. The transmissibility of HFMD is different between male and female individuals. Male cases seem to be more transmissible than female.

## Data

Additional data are available upon emailing to the first author (Yuxue Liao, 42332570@qq.com) on reasonable request.
